# Ethnic heterogeneity in the determinants of HIV/AIDS stigma and discrimination among Nigeria women

**DOI:** 10.1186/s12889-018-5668-2

**Published:** 2018-06-19

**Authors:** Clifford O. Odimegwu, Olatunji Alabi, Nicole De Wet, Joshua O. Akinyemi

**Affiliations:** 1 Demography and Population Studies Program, School of Public Health and Social Sciences, University of the Witwatersrand, Johannesburg, South Africa; 2 Demography and Social Statistics, Federal University, Birnin Kebbi, Kebbi State Nigeria; 3 Department of Epidemiology and Medical Statistics, College of Medicine, University of Ibadan, Ibadan, Nigeria

**Keywords:** HIV/AIDS, Stigma, Discrimination, Ethnicity, NDHS, Nigeria

## Abstract

**Background:**

Stigma and discrimination remains a barrier to uptake of HIV/AIDS counselling and treatment as well as effective HIV reduction programmes. Despite ethnic diversity of Nigeria, studies on determinants of HIV stigma incorporating the ethnic dimension are very few. This paper provides empirical explanation of the ethnic dimension of determinant of HIV stigma and discrimination in Nigeria.

**Methods:**

Nationally representative data from Nigerian Demographic and Health Survey 2013 (Individual recode) was analysed to explore ethnic differentials and homogeneity in the determinants of HIV/AIDS stigma and discrimination among women in multi-ethnic Nigeria.

**Results:**

Result shows that determinants of HIV stigma and discrimination varies by ethnicity in Nigeria. Significant ethnic differentials in HIV/AIDS stigma and discrimination by Secondary school education exist among Hausa and Igbo respectively (OR = 0.79; CI: 1.49-2.28 and OR=1.62; CI: 1.18-2.23, *p*<0.05). Wealth status significantly influenced HIIV/AIDS stigma and discrimination among Hausa, Igbo and Yoruba ethnic groups (*p*<0.05). Knowledge of HIV/AIDS was significantly associated with lower odds of discriminating attitudes among the Hausa and Fulani ethnic groups (OR = 0.45; CI: 0.30-0.67 and OR=0.36; CI: 0.16-0.83, *p*<0.05).

**Conclusions:**

Identifying ethnic differential and homogeneity in predictors of HIV/AIDS stigma is key to reducing HIV/AIDS prevalence in Nigeria and countries with similar settings.

## Background

Reduction in the prevalence and incidence of Human Immunodeficiency Virus (HIV) infection and Acquired Immune Deficiency Syndrome (AIDS) remains high priority among world countries. This became more evident in the resolution to end the AIDS epidemics by the year 2030 within the Sustainable Development Goals 3 (SDGs) target 3.3 [1]. Achieving this goal is anchored on the assumption that, more people will be tested and uptake of HIV counselling and treatment (HCT) will increase through the use of fast-track approach. The approach led to almost a third increase in number of person living with HIV (PLHIV) on antiretroviral therapy from 7.5 million in 2010 to 17million in 2015 [2]. Despite the gains, there were 2.1 million new HIV infection worldwide in 2015 and 150,000 of them were children (less than 15 years) infected through mother-to-child-transmission (MTCT) and lived in sub-Saharan Africa [2].

In Nigeria, there was an estimated 3.2 million PLHIV in 2011 [3]. Thus, Nigeria was ranked as the second highest HIV burden country in the world. Although, trends in national HIV prevalence showed an increase from 1.8% to 5.8% from 1991 to 2001 and a reduction from 5.8% to 3% in 2014 [4], there was an estimated 56, 681 annual HIV positive births and 192,000 annual AIDS death in Nigeria in 2010 [5]. State-wise, the prevalence was as low as 0.9% in Zamfara State and as high as 15.4% in Benue State [4]. HIV epidemiology in Nigeria indicates that infections are higher among women [6]. Evidence suggests that women suffers higher degree of stigma and discrimination than men [7]. There is also variations in prevalence rates by regions. North-central zone had HIV prevalence of 5.8% while North-west had 1.9% and in the southern region, South south and South east had 4.9% apiece while South west had 2.4% [4].

Sustaining the global and national reduction in the prevalence of HIV/AIDS may however be slowed down due to the prevalence of stigma and discrimination. Stigma and discrimination was identified as a major challenge towards achieving universal access to HIV prevention, treatment, care and support [8]. Stigma prevents people from accessing HIV/AIDS counselling talk less of testing to know their status and treatment uptake. Lepine, Terris-Prestholt [9] cited non uptake of testing to ascertain HIV status as one of the biggest obstacle to Nigeria’s efforts at examining new HIV preventions approaches such as Pre-Exposure Prophylaxis for HIV (PrEP) and Treatment as Prevention (TasP) for HIV serodiscordant couples. The authors cited that only 6% of Nigerian couples know their HIV status. Apart from this, stigma related problems has led to broken relationship and non-disclosure of HIV status [10]. The social and psychological effects of stigma and discrimination against PLHIV is so enormous and ranges from loneliness due to isolation (avoidance) from family, friends and colleagues, low self-esteem, compromised treatment, care and support among others [11].

Review of interventions to reduce HIV/AIDS stigma and discrimination shows four major strategies. These are, information-based approaches (which include HIV information in print media, radio jingles/drama etc.), skills building (group learning session to reduce stigma and discrimination), counseling/support for PLHIV and contact with affected group [12]. The authors reported a considerable progress in the HIV stigma-reduction efforts but suggested a need for a comparative analysis of various strategies for economically viability, result advantaged and effective scale up options especially for developing countries.

In Nigeria, government at various levels are working mostly with donor funded programme to implement interventions aimed at reducing HIV stigma and discrimination. The national agency saddled with the responsibility of HIV/AIDS control- National Agency for the Control of AIDS (NACA) – attested to the fact that efforts at ending the epidemic by 2030 may remain a mirage if stigma reduction is not given adequate attention [13]. Nigeria in a bold step towards HIV/AIDS stigma and discrimination reduction passed into law the anti-discrimination act, 2014 which was meant to address the issues of rights and dignity of PLHIV [13]. However less than 1 percent of the States have passed the law in the country. Beyond this, NACA developed the document on HIV/AIDS stigma reduction strategy with the goal of eliminating all form of stigma and discrimination against PLHIV and people directly or indirectly affected by the disease by the year 2020 [13]. Nine strategic objectives which ranges from strengthening media, art and entertainment industry to deliver stigma reduction messages, strengthening the identification process of behavioral, biomedical and structural drivers of HIV related stigma and discrimination and integration of stigma and discrimination activities into the exiting community-based HIV/AIDS programme among others were highlighted. Of all the strategies, media-based HIV stigma and discrimination strategy, seems to be the most effective. Babalola, Fatusi [14] found that exposure to media-based HIV programme resulted in improved attitudes towards PLHIV. However, as robust as the stigma reduction strategy document, ethnic coloration of stigma and discrimination was not adequately addressed.

Studies have documented that stigmatization cut across countries of the globe without exception [15]. Studies conducted in Nigeria on HIV/AIDS stigma and discrimination have documented mostly the determinants of HIV/AIDS stigma and discrimination without measuring the ethnic dimension of the determinants. For instance studies have documented that higher level of education and higher knowledge of HIV were associated with lower level of stigma and discrimination against PLHIV [16–18]. The question here is that is this true for all ethnic groups? Few studies [11, 19] that have examined the ethnic dimension of the determinants of HIV stigma and discrimination were not nationally representative. For instance, Odimegwu, Adedini [11] used mixed method approach to explore the association between stigmatizing and discriminating against PLHIV and uptake of voluntary counselling and treatment (VCT) in two major ethnic groups (Igbo and Yoruba) in Nigeria and found that the Yorubas had higher likelihood of uptake of VCT compared to the Igbos.

Ethnic consideration in policies and interventions, especially in multi-ethnic countries, is very important. Parker [20] suggests that historical events or circumstances influenced the timing and patterns of stigmatization. Loutfy, Logie [21] found cultural or ethnic undertone in the experience of stigma and discrimination by PLHIV in Canada. Nigeria is distinctively diverse in terms of cultures and belief systems. Effects of ethnic differentials and homogeneity pervades almost all issues affecting population, socio-economic and political development in ethnically plural countries [22]. Thus the impact of ethnic differentials in informing interventions and policies to address HIV/AIDS stigma and discrimination cannot be over emphasized [23].

This paper explores the similarities and differences in the determinants of HIV/AIDS stigma and discrimination across the ethnic groups in Nigeria. It is very important to consider the ethnic dimension of the determinants of HIV/AIDS stigma and discrimination in order to inform design of effective interventions for successful HIV/AIDS prevention and treatment programs in Nigeria.

## Methods

### Study area

Nigeria is made up of 36 States and the Federal Capital Territory (FCT) divided into six geo-political zones with visible ethnic diversities. Nigeria has over 250 ethnic groups with Hausa, Igbo and Yoruba constituting the largest groups [24]. Ethnic diversity has been demonstrated in other health-related outcomes in Nigeria [25–27].

### Data Analysis

#### Data Sources

We analysed the Nigeria Demographic and Health Survey (NDHS) individual recode data set of 2013. Data was collected on several issues including knowledge of and attitudes toward HIV/AIDS and other sexually transmitted infections (STIs) from 38,948 women aged 15-49 years [24]. In this paper, analysis was however restricted to 36,064 women aged 15-49 who reported they have ever heard of HIV/AIDS. The details on the sampling frame, sampling technique and sample selection methods used in the survey have been documented elsewhere [24].

### Variable measurement

The dependent variable was stigma and discrimination measures. This was measured as public and self-stigma as postulated by Watson and Corrigan (2001) and used in previous study of HIV stigma in Nigeria [19]. This paper however, because of the nature of the HIV/AIDS questions in the dataset, focused on the public stigma which is measured in three ways: negative belief about the group of people living with HIV/AIDS (stereotypes); agreement with the belief or negative emotional reaction (Prejudice: fear of accorded stigma) and; behavioural response to prejudice (discrimination). Variables aggregated to measure stereotypes (negative belief about PLWHA) include: people with HIV should be ashamed of themselves and people with HIV should be blamed for bringing disease to community. Fear of accorded stigma was measured by whether or not respondent would want HIV infection in the family to remain a secret. Discrimination was measured by aggregating response to the following attitudinal items: willingness to care for relative with AIDS, opinion on if a female teacher infected with HIV, but is not sick, should be allowed to continue teaching, whether or not respondent would buy vegetables from a vendor with HIV etc. Response to these measures were dichotomised into Yes (1) and No (0).

The independent variable was the ethnic affiliation of respondents. This paper goes beyond the contemporary classification of ethnic groups into three major ethnic groups of Hausa, Igbo and Yoruba in Nigeria. The classification included Fulani as separate from Hausa ethnic group. The inclusion of Fulani ethnic groups was based on some of the recent instances where Fulanis are renouncing the “Hausa-Fulani” “label” [28] citing differences in living arrangement and main economic activity (Pastoralist) among others as reasons. Furthermore, this was included separately based on the authors understanding and experience of the cultural dynamics (having worked in rural northern region for over 6 years) of the two ethnic groups (that is Hausa and Fulani).

We also controlled for demographic and socio-economic variables (like education, knowledge of HIV/AIDS, age, residency, occupation, religion etc.) that have been found to influence HIV/AIDS stigma and discrimination [16, 29].

### Analysis

The analysis was done at three levels using STATA 12 software. Descriptive analysis using percentages was used to explore the distribution of respondents by selected demographic and socio-economic characteristics. At the second level of analysis, relationship between ethnic affiliation, explanatory variables and stigma and discrimination measures was analysed using chi-square test of association. At the multivariate level of analysis, binary logistic regression was used to explore the ethnic differentials and homogeneity in determinants of HIV stigma and discrimination in Nigeria. The choice of binary logistic regression was based on the dichotomous nature of the dependent variables measured (stigma and discrimination measures i.estereotype, prejudice and discriminating behaviour explained under variable measurement section above).

## Result

Table 1 shows the descriptive statistics of the respondents by selected characteristics and ethnic group. Highest proportion of the women were aged between 15-24 years cross the four ethnic groups. In terms of distribution of respondents by educational level, majority of women from Hausa and Fulani ethnic groups had no education (72.4% and 83.6% respectively). More than half of the respondents from Igbo and Yoruba ethnic groups had secondary level of education (59.2% and 57.7% respectively). Furthermore, distribution of respondents by place of residence shows that majority (70% and 87% respectively) of the respondents from Hausa and Fulani ethnic groups resides in rural area while the reverse is the case among respondents from Igbo and Yoruba ethnic groups.

**Table 1:** Socio-demographic distribution of respondents by Ethnic group, NDHS 2013

Characteristics	Hausa		Igbo		Yoruba		Fulani	
	Frequency	Percent	Frequency	Percent	Frequency	Percent	Frequency	Percent
Age group								
15-24 years	3761	36.7	2,016	36.2	1,794	33.7	745	37.9
25-34 years	3,355	32.8	1,824	32.7	1,726	32.4	608	31.0
35-44 years	2,124	20.7	1,230	22.1	1,334	25.0	423	21.5
45 years and above	1,003	9.8	504	9.0	475.00	8.9	188	9.6
Level of Education								
No education	7411	72.4	244	4.4	204	3.8	1,642	83.6
Primary	1,243	12.1	1,043	18.7	1,011	18.9	131	6.7
Secondary	1,418	13.8	3,300	59.2	3,071	57.7	154	7.8
Higher	171	1.7	987	17.7	1,041	19.6	37	1.9
Place of residence								
Urban	3,069	30.0	4,105	73.7	4,229	79.4	252	12.8
Rural	7,174	70.0	1,469	26.3	1099.00	20.6	1,712	87.2
Religion								
Christian	87	0.9	5,475	99.7	3,015	57	3	0.1
Islam	10,101	99.1	16	0.3	2,254	43	1,947	99.9
Occupation								
Not working	4,044	39.6	1,891	34.0	1,363	25.6	1120	57.1
Professional/clerical	4,545	44.5	2635.00	47.3	3,305	62.1	565	28.8
Agriculture	81	0.8	707	12.7	142	2.7	60	3.1
Manual	1,536	15.1	332	6.0	513	9.6	216	11.0
Wealth Status								
Poor	6,430	62.8	734	13.2	158	3.0	1,584	80.7
Middle	1,736	16.9	1,156	20.7	569	10.7	168.00	8.6
Rich	2,077	20.3	3,685	66.1	4601.0	86.4	211	10.8
Frequency of exposure to radio								
Not at all	4,106	40.1	950	17.1	492.0	9.2	1,141	58.1
Less than once a week	2,638	25.8	2,106	37.8	1205.0	22.6	316	16.1
At least once a week	3,499	34.2	2,518	45.2	3631.0	68.2	507	25.8
HIV Knowledge								
Poor Knowledge	2,379	23.2	1,415	25.4	1,647	30.9	740	37.7
Good Knowledge	7,863	76.8	4,159	74.6	3,681	69.1	1,224	62.3
Stigma: negative belief about the group								
Disagree	2,659	26.0	3,459	62.1	2,868	53.8	453	23.1
Agree	7,583	74.0	2,116	37.9	2,460	46.2	1,511	76.9
Stigma fear: want hiv infection in family remain a secret?								
No	3,360	32.8	2,076	37.2	2,463	46.2	816	41.5
Yes	6,883	67.2	3,498	62.8	2,865	53.8	1,148	58.5
Discriminating behaviour								
Not discriminating	10,081	98.4	5,425	97.3	5,268	98.9	1,938	98.7
Discriminating	162	1.6	149	2.7	60	1.1	26	1.3

Knowledge of HIV was generally high among respondents from all the four ethnic groups (76.8%, 74.6%, 69.1% and 62.3% among Hausa, Igbo, Yoruba and Fulani respectively). The measures of stigma and discrimination varies across the ethnic groups. Expression of negative belief about PLHIV was lower among Igbo and Yoruba compared to Hausa and Fulani. More than 6 in every 10 of respondents from Hausa and Igbo ethnic groups would want HIV infection remains a secret in the family compared to 5 in 10 respondents among Yoruba and Fulani ethnic groups. Discriminating behaviour was however very low among the respondents across all the ethnic groups.

Table 2 presents the bivariate relationship between selected variables and stigma and discrimination measures by ethnic group. Across the ethnic groups, education and wealth status was significantly associated with negative belief about PLHIV although at different level of statistical significance. For instance, among Hausas, Igbos and Yorubas (*p* <0.001) while among the Fulanis (*p*<0.05). Further, 68.4% of Hausa and 78.3% of Fulani women from poor households had negative belief about PLHIV. However, among the Igbo and Yoruba women, 51.8% and 78% of women respectively from rich households had negative belief about PLHIV. Respondents’ level of education is significantly associated with negative belief about PLHIV among all the ethnic groups. However, educational level was associated with fear of accorded stigma among the Igbos and Yoruba but not among Hausa and Fulani.

**Table 2:** Bivariate relationship between selected characteristics and stigma and discrimination measures by ethnic group, NDHS, 2013

	Hausa			Igbo			Yoruba			Fulani		
	Stigma measures			Stigma measures			Stigma measures			Stigma measures		
Characteristics	Stereotype (*N*=6,752)	Fear of stigma (*N*=5,951)	Discrimination (*N*=135)	Stereotype (*N*=2,110)	Fear of stigma (*N*=3,424)	Discrimination (*N*=149)	Stereotype (*N*=2,392)	Fear of stigma (*N*=2,894)	Discrimination (*N*=45)	Stereotype (*N*=1,430)	Fear of stigma (*N*=1,095)	Discrimination (*N*=33)
**Age group**				***	***		**	***			*	
15-24 years	35.5	36.4	35.6	40.5	37.0	36.2	37.3	36.4	40.0	38.0	40.4	42.4
25-34 years	33.6	33.1	39.3	27.6	32.4	31.5	28.6	31.9	24.4	30.7	30.0	42.4
35-44 years	21.0	21.1	14.8	21.3	22.4	24.8	23.5	23.7	20.0	21.7	20.9	15.2
45 years and above	9.9	9.4	1.6	10.6	8.2	7.4	10.6	8.1	15.6	9.6	8.8	0.0
**Level of Education**	***		*	***	***		***	**		**		***
No education	77.0	73.6	61.5	7	2.9	4	6.4	4.5	6.7	82.9	78.9	45.5
Primary	11.1	11.7	17	24.7	16.7	18.1	22.9	16.6	11.1	7.1	8.5	30.3
Secondary	10.6	12.8	18.5	59.5	61.6	57.7	59.8	57.2	51.1	8.1	10.2	18.2
Higher	1.3	1.9	3	8.8	18.8	20.1	10.9	21.7	31.1	1.8	2.4	6.1
**Place of residence**	***	**	*		***	*	***	***		*		
Urban	23.4	26.4	37.8	68.4	64.8	78.5	71.6	79.3	82.2	14.3	15.8	24.2
Rural	76.6	73.6	62.2	31.6	35.2	21.5	28.4	20.7	17.8	85.7	84.2	75.8
**Religion**	***						***	***				
Christian	0.7	1.3	2.2	99.7	99.6	100	56.7	58.9	53.5	0.3	0.3	0.0
Islam	99.3	98.7	97.8	0.3	0.4	0	43.3	41.1	46.5	99.7	99.7	100
**Occupation**		***		***	***	**	***	**				
Not working	39.3	41.1	43.7	33.2	38.2	20.3	28.7	29.2	28.9	55	56.7	48.5
Professional/clerical	44.6	43.2	44.4	41	45.2	58.8	56.3	58.3	64.4	30.1	28.7	27.3
Agriculture	0.8	0.8	1.5	19.3	11.3	16.2	4.9	2.5	4.4	2.4	1.7	3
Manual	15.3	14.9	10.4	6.5	5.4	4.7	10.2	10	2.2	12.5	12.9	21.2
**Wealth Status**	***		***	***	***	**	***	***		***		**
Poor	68.4	63.8	43	21.9	10.4	20.1	4.7	2.7	4.4	78.3	74.5	48.5
Middle	15.7	16.8	28.9	26.3	19.5	28.9	17.3	11.8	15.6	9.9	10.8	27.3
Rich	15.9	19.4	28.1	51.8	70.1	51	78	85.5	80	11.9	14.7	24.2
**Frequency of exposure to radio**	***	***	***	***	***	**	***	**		***	***	
Not at all	44.7	39.5	32.6	21.7	19.8	7.4	10.4	10.4	13.3	55.9	53.6	42.4
Less than once a week	23.2	28.2	41.5	35.0	33.6	36.2	27.4	21.9	20.0	14.6	17.6	24.2
At least once a week	32.1	32.3	25.9	43.4	46.6	56.4	62.2	67.7	66.7	29.5	28.8	33.3
**HIV Knowledge**	***	***	***	***	***		***				*	
Poor Knowledge	25.2	22.2	38.5	34.4	21.3	30.9	33.9	29.9	24.4	40.1	36.8	54.6
Good Knowledge	74.8	77.8	61.5	65.6	78.7	69.1	66.1	70.1	75.6	59.9	63.2	45.4

*** statistically significant (*p*=0.000), **= Statistically significant (*p*=0.001), *= statistically significant (*p*<0.05)

Knowledge of HIV/AIDS was significantly associated with all the stigma and discrimination measures among Hausas (*p*<0.001) while among the Igbos, HIV/AIDS knowledge was significantly associated with negative belief about PLHIV and fear of accorded stigma and not with discriminating behaviours. Among the Yorubas, HIV/AIDS knowledge was significantly associated with negative belief about PLHIV while among the Fulanis, it was associated with fear of accorded stigma.

Residency (measured by place of residence) was significantly associated with negative belief about PLHIV among Hausas, Yorubas and Fulanis. For instance, more rural women than urban women from Hausa and Fulani ethnic groups 76.6% vs 23.4% and 85.7% vs 14.3% respectively had negative belief about PLHIV. On the other hand, more urban residents than rural residents from Yoruba ethnic group (71.6% vs 28.4%), had statistically significant negative belief about PLHIV. However, residency was not significantly associated with negative belief against PLHIV among the Igbos. In the same vein, residency was significantly associated with fear of accorded stigma among Hausas, Igbos and Yorubas. More rural Hausa women than urban women (62.2% vs 37.8%) compared to more urban Igbo women than rural women (78.5% vs 21.5%) showed discriminating behaviour.

Table 3 shows the results from logistic regression models of the relationship between the selected variables and stigma measure (negative belief about PLHIV) by ethnic group. The odds of having negative belief about PLHIV significantly reduced with higher level of education among Hausa, Igbo and Yoruba ethnic groups. That is, the higher the level of education, the lesser the likelihood of blaming PLHIV for their HIV status among Hausa, Igbo and Yoruba women. Also, Hausa and Igbo women from rich households respectively had 66% and 56% lower odds of having negative belief about PLHIV compared to women from poor households. However, heterogeneity in determinants of stigma was observed among the ethnic groups in terms of the effect of religion and age. For instance, there is a significantly reduced odds of having negative belief as age increases among Igbo and Yoruba ethnic group only. Being a Muslim and residing in rural area was significantly associated with higher odds of having negative belief about PLHIV among Hausa and Yoruba ethnic groups only. However, none of the selected variables was statistically significant associated with having negative belief about PLHIV among the Fulanis.

**Table 3:** Logistics regression of determinants of negative belief about PLWHA by Ethnic group, NDHS 2013

	Hausa		Igbo		Yoruba		Fulani	
	Stigma measures (Stereotype)							
Characteristics	OR	CI, 95%	OR	CI, 95%	OR	CI, 95%	OR	CI, 95%
Age group								
15-24 years	1	[1]	1	[1]	1	[1]	1	[1]
25-34 years	1.04	[0.92, 1.18]	0.74**	[0.63, 0.86]	0.88	[0.74, 1.04]	0.87	[0.67, 1.13]
35-44 years	0.94	[0.81, 1.09]	0.68**	[0.57, 0.82]	0.85	[0.71, 1.02]	0.94	[0.69, 1.27]
45 years and above	0.98	[0.81, 1.19]	0.68*	[0.53, 0.87]	0.94	[0.75, 1.19]	0.78	[0.53, 1.15]
Level of Education								
No education	1	[1]	1	[1]	1	[1]	1	[1]
Primary	0.77*	[0.66, 0.90]	0.97	[0.72, 1.31]	0.87	[0.64, 1.17]	0.63*	[0.42, 0.94]
Secondary	0.56**	[0.48, 0.67]	0.77	[0.56, 1.05]	0.61*	[0.45, 0.81]	0.65	[0.41, 1.02]
Higher	0.52**	[0.37, 0.72]	0.39**	[0.28, 0.56]	0.27**	[0.19, 0.37]	0.35*	[0.16, 0.76]
Place of residence								
Urban	1	[1]	1	[1]	1	[1]	1	[1]
Rural	1.37**	[1.19, 1.57]	0.98	[0.87, 1.12]	1.36**	[1,17, 1.57]	1.04	[0.71, 1.51]
Religion								
Christian	1	[1]	1	[1]	1	[1]	1	[1]
Islam	2.13**	[1.41, 3.23]	0.94	[0.31, 2.87]	1.33**	[1.19, 1.50]		
Occupation								
Not working	1	[1]	1	[1]	1	[1]	1	[1]
Professional/clerical	0.83*	[0.74, 0.94]	1.08	[0.94, 1.26]	0.91	[0.77, 1.07]	1.10	[0.85, 1.41]
Agriculture	0.88	[0.50, 1.57]	1.25*	[1.01, 1.55]	1.49*	[1.02, 2.17]	1.63	[0.70, 3.78]
Manual	0.85*	[0.73, 0.99]	1.23	[0.95, 1.59]	0.94	[0.75, 1.18]	1.12	[0.80, 1.59]
Wealth Status								
Poor	1	[1]	1	[1]	1	[1]	1	[1]
Middle	0.72**	[0.62, 0.84]	0.74*	[0.61, 0.90]	1.17	[0.83, 1.65]	0.62*	[0.43, 0.89]
Rich	0.64**	[0.53, 0.76]	0.55**	[0.46, 0.67]	0.86	[0.62, 1.20]	0.59*	[0.37, 0.95]
Frequency of exposure to Radio								
Not at all	1	[1]	1	[1]	1.0	[1]	1	[1]
Less than once a week	0.78**	[0.68, 0.88]	0.81*	[0.69, 0.96]	1.44*	[1.16, 1.78]	1.14	[0.84, 1.55]
At least once a week	1.11	[0.98, 1.26]	0.89	[0.76, 1.05]	1.0	[0.82, 1.21]	2.94**	[2.14, 4.04]
HIV Knowledge								
Poor Knowledge	1	[1]	1	[1]	1	[1]	1	[1]
Good Knowledge	0.86*	[0.77, 0.98]	0.57**	[0.50, 0.66]	0.82*	[0.72, 0.92]	0.83	[0.66, 1.04]

*** = statistically significant (*p*=0.000), *= statistically significant (*p*<0.05)

Table 4 shows the Odds of HIV/AIDS stigmatization (fear of accorded stigma) among respondents by their ethnic group. Women with secondary education have lower odds of expressing fear of accorded stigma (wanting HIV infection in the family remain a secret) compared to women with no education among Hausa ethnic group while Igbo women with higher level of education have significantly higher odds of expressing fear of accorded stigma than women with no education. Rural residents in Hausa and Igbo ethnic groups also had significantly higher odds of expressing fear of accorded stigma than their urban counterparts in the same ethnic group. However, place of residence was not significantly associated with expression of fear of accorded stigma among Yoruba and Fulani ethnic women.

**Table 4:** Logistics regression of determinants of fear of accorded stigma by Ethnic group, NDHS 2013

Characteristics	Hausa		Igbo		Yoruba		Fulani	
	Stigma measures (fear of accorded stigma)							
	OR	CI, 95%	OR	CI, 95%	OR	CI, 95%	OR	CI, 95%
Age group								
15-24 years	1	[1]	1	[1]	1	[1]	1	[1]
25-34 years	1.01	[0.89, 1.14]	1.02	[0.84, 1.24]	0.95	[0.79, 1.16]	0.73*	[0.56, 0.94]
35-44 years	1.02	[0.88, 1.18]	1.10	[0.87, 1.37]	0.99	[0.81, 1.23]	0.80	[0.59, 1.07]
44 years and above	0.93	[0.77, 1.12]	0.82	[0.62, 1.08]	0.74	[0.56, 0.97]	0.74	[0.50, 1.08]
Level of Education								
No education	1	[1]	1	[1]	1	[1]	1	[1]
Primary	0.91	[0.77, 1.07]	1.48*	[1.05, 2.08]	0.83	[0.59, 1.17]	1.35	[0.88, 2.08]
Secondary	0.73*	[0.61, 0.88]	1.60*	[1.12, 2.28]	0.98	[0.71, 1.37]	1.09	[0.66, 1.78]
Higher	1.07	[0.69, 1.68]	1.53*	[1.03, 2.27]	1.2	[0.84, 1.72]	1.27	[0.56, 2.90]
Place of residence								
Urban	1	[1]	1	[1]	1	[1]	1	[1]
Rural	1.28*	[1.11, 1.49]	1.77**	[1.52, 2.06]	0.90	[0.76, 1.07]	1.03	[0.68, 1.56]
Religion								
Christian	1	[1]	1	[1]	1	[1]	1	[1]
Islam	0.69	[0.39, 1.22]	8.1*	[1.86, 35.26]	1.09	[0.95, 1.25]	1.68	[1.17, 17.12]
Occupation								
Not working	1	[1]	1	[1]	1	[1]	1	[1]
Professional/clerical	0.72**	[0.64, 0.81]	0.81*	[0.67, 0.97]	0.89	[0.74, 1.08]	1.00	[0.78, 1.28]
Agriculture	1.13	[0.63, 1.99]	0.65*	[0.50, 0.84]	0.81	[0.52, 1.25]	0.54	[0.27, 1.05]
Manual	0.85*	[0.73, 0.99]	0.63*	[0.47, 0.85]	0.86	[0.66, 1.12]	1.46*	[0.05, 2.03]
Wealth Status								
Poor	1	[1]	1	[1]	1	[1]	1	[1]
Middle	1.03	[0.88, 1.19]	1.17	[0.94, 1.47]	0.95	[0.62, 1.45]	0.92	[0.62, 1.35]
Rich	1.01	[0.84, 1.23]	2.18**	[1.75, 2.72]	1.50	[0.99, 2.26]	1.01	[0.62, 1.63]
Frequency of exposure to radio								
Not at all	1	[1]	1	[1]	1	[1]	1	[1]
Less than once a week	1.92**	[1.68, 2.20]	0.56**	[0.46, 0.69]	0.68*	[0.53, 0.88]	1.67*	[1.21, 2.30]
At least once a week	1.05	[0.94, 1.19]	0.66**	[0.54, 0..81]	0.70*	[0.55, 0.88]	1.38*	[1.06, 1.80]
HIV Knowledge								
Poor Knowledge	1	[1]	1	[1]	1	[1]	1	[1]
Good Knowledge	1.43**	[1.27, 1.60]	1.37**	[1.17, 1.61]	1.05	[0.91, 1.21]	1.45*	[1.16, 1.80]

*** = statistically significant (*p*=0.000), *= statistically significant (*p*<0.05)

Furthermore, women who were engaged in one occupation or the other had an associated significantly lower odds of expressing fear of accorded stigma than women who were not working among Hausa and Igbo ethnic groups but not among Yoruba and Fulani ethnic group. Among Igbo ethnic groups, women from ‘rich’ households had significantly higher odds of expressing fear of accorded stigma compared to women from ‘poor’ households. Wealth status was not associated with the expression of fear of accorded stigma among Hausa, Yoruba and Fulani ethnic women. Moreover, while having a good knowledge of HIV/AIDS was associated with significantly higher odds of expressing fear of accorded stigma among Hausa, Igbo and Fulani ethnic groups, it was not significant among Yoruba ethnic women.

Determinants of HIV/AIDS discrimination by ethnic group is shown in table 5. The table shows that determinants of discriminating behaviour differs among the ethnic groups in terms of direction of association. Hausa women from ‘rich’ households had higher odds of discriminating behaviour compared with women from ‘poor’ households (*p*<0.05). However, among the Igbo ethnic groups, higher wealth status was associated with significantly lower odds of discriminating behaviour. Also, knowledge of HIV transmission was associated with statistically significant lower odds of expressing discriminating behaviour among Hausa and Fulani ethnic groups but was not significant among Igbo and Yoruba ethnic groups.

**Table 5:** Logistics regression of determinants of discriminating behaviour by Ethnic group, NDHS 2013

Characteristics	Hausa		Igbo		Yoruba		Fulani	
	Discrimination							
	OR	CI, 95%	OR	CI, 95%	OR	CI, 95%	OR	CI, 95%
Age group								
15-24 years	1	[1]	1	[1]	1	[1]	1	[1]
25-34 years	1.47	[0.94, 2.30]	0.73	[0.44, 1.22]	0.49	[0.22, 1.07]	1.21	[0.52, 2.84]
35-44 years	0.80	[0.44, 1.43]	0.74	[0.41, 1.33]	0.54	[0.22, 1.30]	0.97	[0.31, 3.03]
45 years and above	1.62	[0.80, 3.31]	0.79	[0.36, 1.72]	1.64	[0.50, 5.41]		
Level of Education								
No education	1	[1]	1	[1]	1	[1]	1	[1]
Primary	1.16	[0.68, 2.01]	0.72	[0.24, 2.15]	0.69	[0.13, 3.64]	5.27*	[1.77, 15.63]
Secondary	0.99	[0.54, 1.83]	1.25	[0.38, 4.11]	0.97	[0.21, 4.49]	1.92	[0.43, 8.57]
Higher	1.19	[0.36, 3.97]	1.92	[0.55, 6.75]	2.51	[0.53, 11.94]	5.97	[0.81, 44.20]
Occupation								
Not working	1	[1]	1	[1]	1	[1]	1	[1]
Professional/clerical	0.81	[0.54, 1.24]	2.81**	[1.65, 4.78]	0.66	[0.33, 1.32]	1.11	[0.45, 2.74]
Agriculture	1.97	[0.35, 11.03]	1.8	[0.79, 4.11]	1.29	[0.27, 6.18]	3.06	[0.55, 17.05]
Manual	0.43*	[0.22, 0.84]	1.54	[0.60, 3.93]	0.22	[0.03, 1.69]	3.21*	[1.11, 9.24]
Wealth Status								
Poor	1	[1]	1	[1]	1	[1]	1	[1]
Middle	2.90**	[1.76, 4.78]	0.72	[0.39, 1.31]	1.3	[0.24, 7.18]	2.72	[0.93, 7.92]
Rich	2.36*	[1.29, 4.30]	0.33*	[0.18, 0.63]	0.7	[0.15, 3.31]	1.52	[0.36, 6.41]
Frequency of exposure to radio								
Not at all	1	[1]	1	[1]	1	[1]	1	[1]
Less than once a week	2.21*	[1.40, 3.49]	2.57*	[1.10, 5.99]	1.4	[0.42, 4.46]	2.01	[0.67, 6.03]
At least once a week	0.74	[0.45, 1.25]	3.44*	[1.48, 8.01]	1.8	[0.60, 5.13]	1.28	[0.49, 3.35]
HIV Knowledge								
Poor Knowledge	1	[1]	1	[1]	1	[1]	1	[1]
Good Knowledge	0.45**	[0.30, 0.67]	0.81	[0.54, 1.23]	0.74	[0.33, 1.65]	0.36*	[0.16, 0.83]

*** = statistically significant (*p*=0.000), *= statistically significant (*p*<0.05)

## Discussion

The purpose of this study was to examine ethnic variation in the determinants of HIV/AIDS stigma and discrimination among women in Nigeria. The findings demonstrated that ethnic diversities exist among the determinants of HIV/AIDS stigma and discrimination among women in the four ethnic groups (Hausa, Igbo, Yoruba and Fulani) in Nigeria. For instance, it was evidently clear that the higher the level of education, the lower the odds of having a negative belief about PLHIV. This reflect the role of education in reducing stigma against PLHIV. This might be due to the saying that, education, even if it does not make one good, makes it easier to be governed and that with the right education, people tends to understand and demystify the myths about HIV/AIDS transmission and treatment. Mostly, having negative belief abut PLHIV was borne out of the poor initial understanding of the means of contacting the virus which was mostly believed to be through heterosexual intercourse especially in developing countries [30].

Generally, while women in three of the ethnic groups (Hausa, Igbo and Yoruba) have some homogeneity in terms of factors influencing HIV/AIDS stigma and discrimination, women from Fulani ethnic group was very distinct. Pattern of influence of the determinants of HIV/AIDS stigma and discrimination among Fulani women differs from the patterns among women of other ethnic groups and even different among Hausa women which is the most similar and often grouped together in most previous studies that interrogated ethnicity in Nigeria. For instance, considering determinants of HIV/AIDS stigma and discrimination among women from the four ethnic group, findings show that most of the explanatory variables were not significantly associated with stigma and discrimination measures among the Fulani women. The only explanatory variables that were largely associated with stigma and discrimination measures among Fulani women were knowledge of HIV/AIDS, frequency of listening to radio and education. This suggests that there may be other factors at play which might not have been captured in the data. Further studies using anthropological techniques such as ethnography might provide deeper understanding about the beliefs, norms and practices about HIV and AIDS among the Fulanis.

Furthermore, there was a mixed effect in the association between frequency of listening to radio on fear of accorded stigma among the women. For instance, among the Igbo and Yoruba women, the more the frequency of listening to radio, the lower the odds of expressing fear of accorded stigma. On the other hand, among Hausa and Fulani women, the higher the frequency of listening to radio, the higher the odds of expressing fear of accorded stigma. This pattern may be explained by possible lower level of education among Hausa and Fulani women compared to women from Igbo and Yoruba ethnic group in terms of comprehension and interpretation of HIV messages. This may also be due to language challenge in communication especially in translating English language jingles and messages to Hausa language. Also, it was found that good knowledge of HIV/AIDS does not translates to reduced stigma in terms of fear of accorded stigma among the ethnic groups. Knowledge of HIV/AIDS was associated with significantly higher odds of expression of fear of accorded stigma among women of different ethnic groups. This point to the fact that regardless of the level of knowledge of HIV/AIDS, the disease remains dreaded and the improved knowledge have not been able to wane out the initial perception and belief about the disease. Programmes aimed at reversing this association is urgently required in this regard.

In terms of heterogeneity of the HIV/AIDS stigma and discrimination factors, it was found out that while improved wealth status increased the odds of discriminating behaviour among Hausa women, it was associated with reduced odds of discriminating behaviour among women of Igbo ethnic group. This may be due to the fact that among the Hausa- where there is a visible stack difference between the wealthiest and the poorest- the disease may be perceived as the disease of the poor. Also, difference in level of education among the ethnic groups (Hausa vs Igbo) may explain this finding.

With the evidence from the findings, previous studies suggesting ethnic differentials and homogeneity in determinants of HIV/AIDS stigma and discrimination were confirmed. Rao, Pryor [20, 31] found out that PLWHA experiences stigma and discrimination based on their ethnic background and that the form and period in which stigma appeared were influenced by the historical circumstances thus, suggesting ethnic or cultural differentials in determinants of stigma and discriminating attitudes. In Nigeria, few studies [11, 29, 32] opined that elements of ethnic differentials do exist in HIV/AIDS stigma and discrimination in Nigeria. For instance, Adebayo, Anyanti [29] posits that accepting attitudes towards PLWHA varies across different ethnic groups. Going by this, we argued that for optimal HIV/AIDS VCT uptake and eventual reduction in HIV/AIDS prevalence in Nigeria, interventions incorporating and sensitive to ethnic and cultural diversity of Nigeria should be deployed. Evidence-informed policies from such interventions that works at various ethnic division should be put in place if the scourge of the disease would be reduced in Nigeria. Furthermore, more studies especially qualitative studies on ethnic differentials and homogeneity in determinants of HIV/AIDS stigma and discrimination should be conducted for detailed understanding of ethnic or cultural definitions of stigma and discriminating attitudes. Understanding this within the ethnic context and its consequences for affected individuals and communities can help us develop better approaches for combating this phenomenon and reducing its effects.

## Conclusion

This paper set out to apply ethnic lens to understanding differentials and homogeneity in the determinants of HIV/AIDS stigma and discrimination in a multi-ethnic sensitive Nigeria. Findings show the existence of ethnic differentials and homogeneity in the determinants of HIV/AIDS stigma and discrimination in Nigeria. To this extent, we conclude that one single policy document at addressing HIV/AIDS stigma and discrimination with a view to in the short term, improve the uptake of HIV/AIDS VCT might not yield expected result. In the long run, the reduction in the prevalence of HIV/AIDS in particular stigma and discrimination induced ones, might not be easily achieved. Interventions and policies that incorporate various ethnic and cultural concerns is needed to achieve the goal of zero discrimination.

### Limitation

This study was limited to four major ethnic groups and may not be representative of the various minority ethnic groups in Nigeria. Despite these, the study is a modest contribution to the scarce literature on ethnic perspective of HIV/AIDS stigma and discrimination in Nigeria.

## Abbreviations

HIV: Human immunodeficiency virus
AIDS: Acquired Immune deficiency syndrome
SDGs: Sustainable development goals
HCT: HIV counselling and treatment
PLHIV: Person living with HIV
MTCT: mother-to-child-transmission
PrEP: Pre-exposure prophylaxis for HIV
TasP: Treatment as prevention
NACA: National Agency for the Control of AIDS
VCT: voluntary counselling and treatment
FCT: Federal capital territory
STI: Sexually transmitted infections
